# Multicenter Retrospective Review of Ketamine Use in Pediatric Intensive Care Units (Ketamine-PICU Study)

**DOI:** 10.1155/2024/6626899

**Published:** 2024-07-27

**Authors:** Christine M. Groth, Christopher A. Droege, Preeyaporn Sarangarm, Michaelia D. Cucci, Kyle A. Gustafson, Kathryn A. Connor, Kimberly Kaukeinen, Nicole M. Acquisto, Sai Ho J. Chui, Deepali Dixit, Alexander H. Flannery, Nina E. Glass, Helen Horng, Mojdeh S. Heavner, Justin Kinney, William J. Peppard, Andrea Sikora, Brian L. Erstad

**Affiliations:** ^1^ Adult Critical Care and Emergency Medicine Department of Pharmacy University of Rochester Medical Center, 601 Elmwood Ave. Box 638, Rochester 14642, NY, USA; ^2^ Department of Pharmacy Services UC Health-University of Cincinnati Medical Center, Cincinnati, OH, USA; ^3^ Emergency Medicine Department of Pharmacy University of New Mexico Hospitals, Albuquerque, NM, USA; ^4^ Department of Pharmacy Cleveland Clinic Akron General, Akron, OH, USA; ^5^ Department of Pharmacy Practice Northeastern Ohio Medical University, Rootstown, OH, USA; ^6^ Department of Pharmacy Practice and Administration St. John Fisher University, Rochester, NY, USA; ^7^ Department of Biostatistics and Computational Biology University of Rochester Medical Center, Rochester, NY, USA; ^8^ Department of Pharmacy University of Rochester Medical Center, Rochester, NY, USA; ^9^ Department of Pharmacy University of Maryland Medical Center, Baltimore, MD, USA; ^10^ Department of Pharmacy Robert Wood Johnson University Hospital, New Brunswick, NJ, USA; ^11^ Department of Pharmacy Practice and Science University of Kentucky College of Pharmacy, Lexington, KY, USA; ^12^ Department of Surgery Rutgers New Jersey Medical School, Newark, NJ, USA; ^13^ Department of Pharmaceutical Services University Hospital of New Jersey, Newark, NJ, USA; ^14^ Department of Pharmacy Practice and Science University of Maryland School of Pharmacy, Baltimore 21201, MD, USA; ^15^ Critical Care Medicine Department of Pharmacy Practice Loma Linda University School of Pharmacy, Loma Linda, CA, USA; ^16^ Department of Pharmacy Froedtert & the Medical College of Wisconsin, Milwaukee, WI, USA; ^17^ Department of Clinical and Administrative Pharmacy University of Georgia College of Pharmacy Augusta University Medical Center, Augusta, GA, USA; ^18^ Department of Pharmacy Practice and Science University of Arizona College of Pharmacy, Tucson, AZ, USA

## Abstract

**Objective:**

Describe continuous infusion (CI) ketamine practices in pediatric intensive care units (PICUs) and evaluate its effect on pain/sedation scores, exposure to analgesics/sedatives, and adverse effects (AEs).

**Methods:**

Multicenter, retrospective, observational study in children <18 years who received CI ketamine between 2014 and 2017. Time spent in goal pain/sedation score range and daily cumulative doses of analgesics/sedatives were compared from the 24 hours (H) prior to CI ketamine to the first 24H and 25−48H of the CI. Adverse effects were collected over the first 7 days of CI ketamine.

**Results:**

Twenty-four patients from 4 PICUs were included; median (IQR) age 7 (1-13.25) years, 54% female (*n* = 13), 92% intubated (*n* = 22), 25% on CI vasopressors (*n* = 6), and 33% on CI paralytics (*n* = 8). Ketamine indications were analgesia/sedation (*n* = 21, 87.5%) and status epilepticus (*n* = 3, 12.5%). Median starting dose was 0.5 (0.48–0.70) mg/kg/hr and continued for a median of 2.4 (1.3–4.4) days. There was a significant difference in mean proportion of time spent within goal pain score range (24H prior: 74% ± 14%, 0–24H: 85% ± 10%, and 25−48H: 72% ± 20%; *p*=0.014). A significant reduction in median morphine milligram equivalents (MME) was seen (24H prior: 58 (8–195) mg vs. 0–24H: 4 (0–69) mg and *p*=0.01), but this was not sustained (25−48H: 24 (2–246) mg and *p*=0.29). Common AEs were tachycardia (63%), hypotension (54%), secretions/suctioning (29%), and emergence reactions (13%).

**Conclusions:**

Ketamine CI improved time in goal pain score range and significantly reduced MME, but this was not sustained. Larger prospective studies are needed in the pediatric population.

## 1. Introduction

Ketamine is an N-methyl-D-aspartate (NMDA) receptor antagonist with increasing use for managing pain and agitation in critically ill pediatric patients [1, 2]. By blocking the neuroexcitatory effects of glutamate on these receptors, it decreases calcium influx to prevent the release of prostaglandins and nitric oxide resulting in a dissociative anesthetic state and analgesia. Its pharmacology is quite complex with additional analgesic effects produced through opioid agonism, decreased serotonin reuptake, and sodium channel blockade [3]. It possesses bronchodilation and antiepileptic properties and therefore has also been used for bronchospasm and status epilepticus (SE) [4–6]. Its place in therapy is not fully understood as data in the pediatric population are limited by small, observational studies that do not evaluate impact on clinical outcomes [2, 7, 8].

Pain and agitation guidelines for critically ill pediatric patients recommend nonopioids and opioids for analgesia, while dexmedetomidine is considered first line for agitation [9]. Other medications frequently used for sedation include benzodiazepines and propofol; however, propofol carries risk of severe metabolic acidosis and benzodiazepines are associated with delirium and increased length of stay, which may limit their use in pediatric patients [2, 10].

Ketamine may be a reasonable alternative to traditional sedatives and analgesics [9]. In contrast to opioids and dexmedetomidine, it is a sympathomimetic known to cause hypertension and tachycardia, which may be beneficial for patients with hemodynamic instability [3, 11]. The limited data available evaluating continuous infusion (CI) ketamine as an adjunctive agent in critically ill pediatric patients does suggest a potential benefit with minimal adverse effects [1, 2, 4, 8, 12–15]. However, several questions remain, including optimal dose, incidence of adverse effects (AEs), impact on pain and sedation endpoints, and risk of withdrawal syndrome.

The study aim was to describe the use of CI ketamine in critically ill pediatric patients, including indications, dose and duration, proportion of time in goal pain and sedation score range, exposure to analgesics and sedatives, AEs, and patient outcomes. We hypothesized CI ketamine would increase time spent in goal pain and sedation score ranges and reduce exposure to other analgesics and sedatives.

## 2. Materials and Methods

This was a multicenter, retrospective, observational study of patients who received CI ketamine in a pediatric intensive care unit (PICU). The primary objective was to describe CI ketamine use (indication, dose, and duration of therapy). Secondary objectives were to determine if CI ketamine improves time spent in goal pain and sedation score range, reduces exposure to other analgesics and sedatives, and describes AEs and patient outcomes.

The ketamine-ICU study group designed and conducted the study. The American College of Clinical Pharmacy (ACCP) Practice-Based Research Network (ACCP Foundation) recruited members and additional sites were recruited to participate in data collection from the ACCP Critical Care Practice and Research Network (PRN) via electronic mail and targeted contact by investigators. All study sites received approval for the conduct of this study with waivers of informed consent from their Institutional Review Boards (IRBs). Each site was listed within the IRB approval from the University of Rochester Office for Human Subject Protection (STUDY00001686), which functioned as the coordinating site. The guidelines for reporting observational studies with the Strengthening the Reporting of Observational Studies in Epidemiology (STROBE) checklist was used to strengthen the reporting of our findings.

### 2.1. Patient Population

Patients were included if <18 years and received CI ketamine for any duration of time while in a PICU between January 2014 and December 2017. Patients were excluded if transferred from an outside hospital already receiving CI ketamine. Participating sites collected data on as many patients as possible starting with the most recent patients first.

### 2.2. Data Collection and Outcomes

A secure Research Electronic Data Capture (REDCap) database was used to perform data collection [16]. The ketamine-ICU study group developed and tested the data collection forms, and the coordinating site hosted conference calls with participating sites to review areas of potential ambiguity, reduce variability in data collection, and ensure data integrity.

Institutional and patient demographics were collected to describe the participating sites and patient population. Ketamine indication, initial bolus doses and infusion rate, titration instructions, CI concentration, daily minimum and maximum infusion rates, duration of therapy, and cumulative doses for the first 7 days of therapy were collected to describe CI ketamine use.

For those receiving CI ketamine for a pain or sedation indication, the time spent in goal pain or sedation score range was compared between the 24 hours (H) prior and CI ketamine initiation to the first 0–24H and 25−48H of the infusion. All pain and sedation scores including the scale used (RASS: Richmond Agitation Scale Score or SAS: Sedation Analgesia Score), the score, the time the score has taken, and whether the score was in the goal range were collected. Goal pain and sedation scores were obtained from the ketamine order titration instructions or from documentation in a chart note. Due to the potential for poor chart documentation, if goal pain and sedation scores were unknown, we assumed they were in the range of mild to no pain and awake to slightly sedated (RASS -2 to 0 or SAS 3-4). Baseline oral/intravenous (IV) analgesics and sedatives given in the 24H prior to ketamine including epidurals were collected. Cumulative doses of IV analgesics (opioids) and sedatives (benzodiazepines, propofol, and dexmedetomidine) given in the 24H prior to ketamine were compared to cumulative doses given in the first 0–24H and 25−48H of the infusion. For comparison, cumulative doses of opioids and benzodiazepines were converted to IV morphine milligram equivalents (MME) in mg (fentanyl 100 mcg = hydromorphone 1.5 mg = morphine 10 mg) and midazolam equivalents in mg (lorazepam 1 mg = diazepam 5 mg = midazolam 2 mg), respectively [17]. Antipsychotic use at baseline and during the first 7 days of CI ketamine was also collected.

Hemodynamic adverse effects (hypertension, hypotension, tachycardia, bradycardia, and cardiac abnormalities) were evaluated in the first 4, 24, and 48H after CI ketamine initiation. Vital signs (systolic blood pressure (SBP), mean arterial pressure (MAP), and heart rate (HR) were compared in the 4H prior to and 4H after CI ketamine initiation. Incidence of seizures, hypertonia, hypersalivation, delirium, and emergence, allergic, and injection site reactions were collected during the first 7 days of CI ketamine or until it was discontinued.

Patient outcomes evaluated in all patients were ICU and hospital length of stay, duration of mechanical ventilation, discharge disposition, and mortality. Additional details on study methods and data collection points including definitions of AEs are previously reported [18].

### 2.3. Statistical Analysis

Data were evaluated using IBM SPSS Statistics for Windows, Version 28.0. Armonk, NY: IBM Corp and SigmaPlot® 14 software (Systat®, San Jose, CA) and reported using descriptive statistics with mean and standard deviation (SD) or median and interquartile range (IQR), as appropriate.

Differences in before and after ketamine data were compared using paired *t*-test, Wilcoxon signed rank test, one-way repeated measures analysis of variance (ANOVA), or one-way ANOVA on ranks depending on the Shapiro–Wilk normality test and number of comparison groups. Differences in hemodynamic variables were assessed using Cochran's Q-test. Median values were used for comparison of integer-based scoring systems (e.g., SAS; RASS). As various different pain and sedation scales were used between institutions, collected values were categorized and evaluated as time within goal based on institution-specific or patient-specific goals at the time of data collection.

## 3. Results

### 3.1. Institution and Study Population Demographics

Four geographically diverse institutions were included. Two had clinical practice guidelines for managing pain and agitation (50%) and only 1 (25%) included ketamine. Details on institution demographics, pain, agitation, and delirium assessment tools and ketamine practices are in the Supplemental Material Table S1.

There were 24 patients evaluated with a median (IQR) age of 7 years (1–13.25) and majority were female (*n* = 13, 54%). Most patients were included from one participating site (*n* = 14, 58%). Additional patient demographics are in Table 1, and individual patient characteristics can be found in the Supplemental Material Table S2.

### 3.2. Ketamine Indication, Dose, and Duration

CI ketamine was used for analgosedation (when used for both analgesia and sedation) (*n* = 12, 50%), sedation (*n* = 8, 33%), SE (*n* = 3, 12.5%), and analgesia (*n* = 1, 4%). Ketamine was more commonly used as an adjunctive agent (*n* = 15, 71%) rather than a first-line agent (*n* = 6, 29%). In patients on CI ketamine for analgesia or sedation, 17 (81%) patients were on additional CI opioids or sedatives and 9 (43%) were on adjunctive nonopioid analgesics and sedatives at baseline. Only 16 (76%) patients were on opioids prior to ketamine. Additional information on baseline analgesic, sedative, and antipsychotic use is in Table 2 and Supplemental Material Table S2.

Half (*n* = 12, 50%) of the study population received an initial ketamine bolus dose and all received weight-based CI ketamine doses in either mg/kg/hr (*n* = 15, 62.5%) or mcg/kg/min (*n* = 9, 37.5%) with actual body weight used most often. After converting units to mg/kg/hr, the median initial and discontinuation rates were 0.5 (0.48–0.7) and 0.56 (0.3–1.3), respectively. Higher starting doses were used for indications of analgosedation, sedation, and SE, then doses used for analgesia alone. The median duration of CI ketamine was 2.4 (1.3–4.4) days. A fixed-rate strategy was used more than a titratable CI (*n* = 13, 54% vs. *n* = 11, 46%). Complete CI ketamine dose information can be found in Table 3 and Supplemental Material Tables S2 and S3.

### 3.3. Pain Scores, Sedation Scores, and Analgesic, Sedative, and Antipsychotic Uses

Pain scores were analyzed only in patients receiving CI ketamine for an analgesia or sedation indication (*n* = 21). In the 24H prior to, first 0–24H, and 25−48H of CI ketamine, pain scores were recorded in 15/21 (71%), 14/21 (67%), and 9/10 (90%) patients who remained on CI ketamine, respectively The median number (IQR) of times a score was recorded during these time frames were 9 (7–9), 10 (6–13), and 3 (2.4–8) times, respectively (*p* < 0.001). Goal pain scores were known in 3 (14%) patients. There was a significant difference in the mean proportion of time spent within goal pain score range after CI ketamine initiation (*n* = 21) (24H prior: 74% ± 14%, 0–24H: 85% ± 10%, 25−48H: 72% ± 20%; *p*=0.014).

Sedation scores were analyzed only in patients receiving CI ketamine for an analgesia or sedation indication (*n* = 21). In the 24H prior to, first 0–24H, and 25−48H of CI ketamine, sedation scores were only recorded in 4/21 (19%), 2/21 (9.5%), and 2/10 (20%) patients who remained on CI ketamine, respectively. The median number (IQR) of times a score was recorded during these time frames were 1 (1-2), 2 (1.5–2), and 1 (1-2) time, respectively (*p*=0.03). Goal sedation scores were known in 3 (14%) patients. Due to the low number of patients with a level of sedation assessment performed, time spent in goal range was not evaluated.

Analgesic, sedative, and antipsychotic uses were analyzed only in patients receiving CI ketamine for an analgesia or sedation indication. Of these, 16 (76%) patients received opioids and benzodiazepines. Analgesic requirements were found to be significantly reduced in the first 24H after the addition of CI ketamine. Median IV MME decreased from 58 (8–195) mg in the 24H prior to ketamine to 4 (0–69) mg in the first 0–24H of the CI, (*p*=0.008). However, this did not remain significant when compared across all three time frames 25−48H: median IV MME 24 (2–246) mg (*p*=0.29). Benzodiazepine use did not significantly change with the addition of CI ketamine. Mean midazolam equivalents were 61 ± 84 mg in the 24H prior to ketamine, 39 ± 55 mg in the first 0–24H, and 68 ± 81 mg in the 25−48H time frame (*p*=0.35). Mean propofol doses increased during each time frame but did not reach statistical significance and was largely driven by one patient that was greater than 70 kg (24H prior: 2303 ± 3929 mg, 0–24H: 2614 ± 3612 mg, and 25−48H: 3230 ± 4449 mg, *p*=0.21). Mean dexmedetomidine doses were also no different across the three time frames (24H prior: 1525 ± 1701 mcg, 0–24H: 1305 ± 1507 mcg, and 25−48H: 1726 ± 1852 mcg, *p*=0.67). Very few patients remained on ketamine (*n* = 10/21, 48%) in the 25−48H time frame which likely affected these outcomes. Also, few patients remained on opioids (*n* = 7/16, 44%), benzodiazepines (*n* = 8/15, 53%), and dexmedetomidine (*n* = 3/7, 43%) during this time frame, while the majority were still on propofol (*n* = 3/4, 75%). Antipsychotic use was low with only one patient receiving both haloperidol and an atypical antipsychotic at baseline. One additional patient was started on quetiapine after CI ketamine initiation.

### 3.4. Hemodynamic Changes and Adverse Effects

Hemodynamic changes before and after CI ketamine were evaluated in all 24 patients and were variable. There was a significant reduction in both median SBP (105 (91–121) mm Hg vs. 97 (86–117) mm Hg, *p*=0.029) and MAP (72 (57–88) mm Hg vs. 66 (54–75) mm Hg, *p*=0.02) after the addition of ketamine, but no change in HR (129 (108–152) vs. 126 (104–156) beats per minute, *p*=0.86). During the initial 4H of CI ketamine hypertension, hypotension, tachycardia, and bradycardia occurred in 4% (*n* = 1), 54% (*n* = 13), 63% (*n* = 15), and 4% (*n* = 1) of patients, respectively. There was no difference in the incidence of hypertension, hypotension, tachycardia, or bradycardia at 5−24H (21%, 54%, 67%, and 4%, respectively) or at 25−48H (25%, 67%, 67%, and 0%, respectively). There were no reports of any cardiac abnormalities such as atrial fibrillation, ventricular tachycardia, myocardial infarction, or heart block.

Adverse effects potentially associated with CI ketamine were evaluated in all 24 patients during the initial seven days of therapy. Increased secretions or suctioning were identified in 7 (29%) patients, of which 6 (86%) were within the first 24H. This did not appear to be severe, as no anticholinergic or mucolytic agents were used in any patients. Additional AEs thought to be related to CI ketamine included hypertonia in one patient, hallucinations in one patient, and QT prolongation which led to discontinuation of ketamine in one patient. Emergence reactions at CI ketamine discontinuation were reported in 3 (13%) patients. One of these patients received an antipsychotic within 4H of CI ketamine discontinuation. Delirium was not assessed or documented in the medical record for any patients.

### 3.5. Patient Outcomes

Patient outcomes were consistent with a severely ill patient population. Median PICU and hospital lengths of stay were 22 days (5–40) and 27 days (7–68), respectively. The majority of patients survived and was discharged home (Table 4).

## 4. Discussion

To our knowledge, this is the first multicenter study describing use of CI ketamine in PICUs across the United States. CI ketamine was safe and most commonly used as an adjunctive sedative agent. Patients had improved time in goal pain score range after addition of CI ketamine with reduced exposure to opioids initially, but the effect on opioid exposure was not sustained. This has important implications on clinical practice as it gives clinicians another pharmacotherapeutic CI option for managing pain and agitation.

The most common indications for CI ketamine in critically ill children found in this study included analgosedation, sedation, SE, and analgesia. This is consistent with another single-center study in 60 PICU patients that found 78% received CI ketamine for analgosedation, 18% for bronchospasm, and 4% for SE [15]. Despite several reports on the use of CI ketamine for the treatment of bronchospasm, we did not have any patients who specifically used CI ketamine for this indication [4, 19–21]. Although, two patients with CI ketamine for analgosedation or sedation were admitted to the PICU for respiratory failure due to asthma. Interestingly, our results also highlight the variability in dosing of CI ketamine and lack of consensus on the use of bolus doses and units used for infusion rates.

In this study, the majority of patients receiving CI ketamine also received opioids or benzodiazepines (76%). A statistically significant decrease in overall MME was found during the first 24H, and a nonsignificant decrease was observed between 25 and 48H following CI ketamine initiation. This could be due to tolerance that develops to analgosedatives over time. Converse to the findings associated with opioids, there was no significant difference in midazolam, propofol, or dexmedetomidine doses after CI ketamine initiation. A meta-analysis in postoperative pediatric patients showed no opioid-sparing effects of ketamine [22]. On the contrary, retrospective studies similar to our population including PICU patients demonstrated CI ketamine significantly reduced opioid requirements [1, 15].

In regards to the impact of CI ketamine on the use of sedative agents, the most robust study was conducted in 60 PICU patients comparing sedatives immediately before initiation to doses at 24H after initiation of CI ketamine [15]. In patients receiving ketamine for sedation, there was no significant changes in doses of benzodiazepines, propofol, or dexmedetomidine, but 81% (*n* = 38) did not require any further increases in doses of these medications. Heiberger et al. found the addition of CI ketamine in those with inadequate sedation reduced need for bolus doses of sedation [2]. Johnson et al. demonstrated decreases in both dexmedetomidine and midazolam doses between CI ketamine initiation and discontinuation and no change in propofol doses; however, this could have been due to decreases in sedation needs, in general, over time [8]. Our study suggests CI ketamine provides more opioid-sparing effects than sedation-sparing effects.

In the present study, SE accounted for only a small portion of the CI ketamine use. The literature evaluating ketamine for this indication is limited to case series and case reports making it difficult to draw meaningful conclusions [5, 23]. Of note, a randomized open-label trial that sought to evaluate the safety and efficacy of CI ketamine in SE in pediatric patients was terminated early due to futility [24].

Ketamine is conventionally thought of as a sympathomimetic with negative inotropic effects, especially in adult patient populations [3]. However, the results from this study appear to challenge this assumption in the pediatric population as there were significant decreases in median SBP and MAP coinciding with CI ketamine initiation. This is similar to findings in other case series assessing hemodynamic effects of ketamine in pediatrics [25]. This study found a 7.7 mm·Hg decrease in SBP, as well as a 5.8 mm·Hg decrease in MAP within the first 4H of CI ketamine initiation. In addition, only one patient was reported as having hypertension compared to 13 patients (54%) with hypotension in the initial 4H of starting ketamine. This may indicate the patient population included in this study was catecholamine depleted where the negative inotropic effects of ketamine may be more predominant over its sympathomimetic effects.

The AEs of ketamine in children are not well established although current studies have reported few AEs associated with CI ketamine administration [2, 7, 8, 15]. An analysis of 22 patients reported no AE while a larger retrospective analysis of 73 patients reported three AE, including hypertension, emergence phenomenon, and one possible episode of iatrogenic withdrawal syndrome (IWS) with CI ketamine use [2, 8]. A second observational analysis of 60 patients reported three AE of hypertension, hypersalivation, and delirium [15]. However, AE and IWS were not included in all the analyses. Our study found similar AEs of hypersalivation and emergence reactions to those previously reported. There were no assessments for delirium making it difficult to draw conclusions on the effect of ketamine on ICU-related delirium.

Since the completion of our study, a single-center, observational, prospective study was conducted evaluating the safety and efficacy of CI ketamine in 77 PICU patients [26]. Overall, their patient population was younger (median age 16 months compared to 7 years in our study) and CI ketamine was used mostly for respiratory and postsurgical cases, with more asthma patients, compared to our study. They used higher CI ketamine doses for a longer duration (15–30 mcg/kg/min for 3.8 days compared to 5-6 mcg/kg/min for 2.4 days in our study) and had higher continuous opioid and benzodiazepine use than our study (99% and 88% vs. 75% and 53%, respectively) with similar propofol and dexmedetomidine use. Both studies demonstrated reduced morphine requirements and improved pain/sedation scores 24 hours after initiating CI ketamine (comfort B score in their study and pain scores in our study). This study reported reduced benzodiazepine requirements, whereas our study did not, which could be attributed to higher use of benzodiazepines in their study. Adverse events were similar with more hemodynamic instability found in our study; however, their study did not specifically look at hypotension. This study did attempt to compare clinical characteristics in those categorized as ketamine responders and nonresponders and found lower PIM3 scores, higher ketamine doses, and greater use of bronchodilators in nonresponders. The authors commented that these patients tended to be on CI ketamine for bronchodilation and were more likely to be paralyzed, therefore assessing efficacy was challenging and can only be hypothesis generating. Though, it does indicate higher doses do not lead to improvements in efficacy and should be weighed against the possible increased risk in adverse effects. Although single center, this study reports effective and safe use of CI ketamine in PICU patients with similar results to our findings.

Our study is novel in that it is the first multicenter study evaluating CI ketamine in critically ill pediatric patients. Major limitations include the small sample size and retrospective design with lack of a comparison group and potential missing or incomplete data. This limits the external validity of these results and may not be generalizable to larger populations. We arbitrarily chose goal levels of pain and sedation if not known in a large percentage of patients and most patients were from a single institution which could introduce bias. Several confounders are likely present in critically ill patients, therefore the hemodynamic effects seen with CI ketamine can only be used to describe the patient population receiving this therapy. Despite these limitations, these data add value to the current literature describing CI ketamine use in a real-world setting and highlight the need for a more standardized approach to its administration.

## 5. Conclusion

In critically ill pediatric patients, there is a lack of large-scale data to inform CI ketamine practices. We found CI ketamine is associated with increased time in goal pain score range and reduced exposure to opioids, although this was not sustained. However, it appears to have an acceptable safety profile. Due to its limitations, these data can only be hypothesis generating and should serve as a call for national PICU databases for studying sedation/analgesia practices and larger prospective studies to evaluate CI ketamine use specifically in pediatric patients to advise current and future practices.

## Figures and Tables

**Table 1:** Baseline demographics for patients receiving continuous infusion ketamine (*n* = 24).

Demographics	Value
Weight (kg), median (IQR) (*n* = 12)	36 (10–69)
Height (inches), median (IQR) (*n* = 9)	60 (45–65)
Ethnicity, *n* (%)	
Non-Hispanic	21 (87)
Hispanic	2 (8)
Unknown	1 (4)
Race, *n* (%)	
White	13 (54)
Black/African-American	5 (21)
Unknown	6 (25)
Primary ICU type, *n* (%)	
Pediatric	22 (92)
Trauma	2 (8)
Admitting diagnosis, *n* (%)	
Respiratory failure	12 (50%)
Seizure	3 (12.5)
Sepsis	3 (12.5)
Post-op elective surgery	2 (8)
Asthma	2 (8)
Trauma	1 (4)
Abdominal perforation	1 (4)
Relevant comorbidities, *n* (%)	
Seizure	4 (17)
Psychiatric illness	2 (8)
Substance abuse	1 (4)
Renal failure	1 (4)
Tachyarrhythmias	1 (4)
None	17 (71)
Mechanical ventilation, *n* (%)	22 (92)
Chronic mechanical ventilation	1 (4.5)
Ketamine initiated prior to intubation	1 (4.5)
Ketamine discontinued after extubation	3 (13.6)
Clinical characteristics, *n* (%)	
Continuous vasopressors	6 (25)
Continuous neuromuscular blockade	8 (33)
FiO_2_ >50%	11 (46)
pH <7.15	3 (24)
GCS, median (IQR)	7.5 (3–14)
Consult service recommending ketamine, *n* (%)	
Yes	4 (17)
Type of consult service, *n* (%)	
Neurology	3 (75)
Pulmonary	1 (25)

IQR: interquartile range; ICU: intensive care unit; FiO_2_: fraction of inspired oxygen; GCS: Glasgow Coma Scale.

**Table 2:** Baseline analgesic, sedative, and antipsychotic uses (*n* = 21).

Analgesics, sedatives, and antipsychotics	Value
*PRN medications*	
IV or PO PRN opioids, *n* (%)	
Fentanyl	6 (29)
Morphine	4 (19)
Hydromorphone	1 (5)
IV or PO PRN sedatives, *n* (%)	
Midazolam	3 (14)
Lorazepam	2 (10)
Diazepam	1 (5)
IV or PO PRN antipsychotics, *n* (%)	
Haloperidol	1 (5)
Atypical antipsychotics	1 (5)
IV or PO PRN medications, none, *n* (%)	9 (43)

*ATC medications*	
IV, PO or transdermal ATC opioids, *n* (%)	
Methadone	1 (5)
IV or PO ATC sedatives, *n* (%)	
Lorazepam	1 (5)
Clobazam	1 (5)
IV or PO ATC medications, none, *n* (%)	18 (86)

*Continuous infusions*	
Continuous infusion opioids, *n* (%)	
Fentanyl	10 (48)
Morphine	3 (14)
Hydromorphone	1 (5)
Continuous infusion sedatives, *n* (%)	
Midazolam	11 (52)
Dexmedetomidine	7 (33)
Propofol	3 (14)
Continuous infusion medications, none, *n* (%)	4 (19)

*Adjunctive nonopioid analgesics and sedatives*	
No adjunctive agent, *n* (%)	12 (57)
Acetaminophen, *n* (%)	8 (38)
Lidocaine, *n* (%)	1 (5)
Baclofen, *n* (%)	1 (5)
Clonidine, *n* (%)	1 (5)

IV: intravenous; PO: by mouth; PRN: as needed; ATC: scheduled around the clock.

**Table 3:** Continuous infusion ketamine dose and administration (*n* = 24).

Dose information	Value
*Loading dose*	
Dose (mg/kg), median (IQR), (*n* = 12)	1.0 (0.98–1.13)
Weight (kg), median (IQR)	28 (12–46)
Weight used, *n* (%)	
Actual	12 (100)

*Continuous infusion*	
Initial units, *n* (%)	
mg/kg/hr	15 (62.5)
mcg/kg/min	9 (37.5)
Initial dose^*∗*^ (mg/kg/hr), median (IQR)	
Overall	0.5 (0.48–0.70)
Analgesia	0.18 (0.18–0.18)
Analgosedation	0.5 (0.39–0.60)
Sedation	0.5 (0.45–1.0)
Status epilepticus	0.6 (0.55–4.8)
Weight (kg), median (IQR)	20 (8–51)
Weight utilized, *n* (%)	
Actual	23 (96)
Adjusted	1 (4)

*Ketamine titratable dose*	
Ketamine-titratable CI, *n* (%)	11 (46)
Initial rate, dose, median (IQR)	
mcg/kg/min (*n* = 3)	5.0 (4.3–7.5)
mg/kg/hr (*n* = 8)	0.2 (0.1–0.5)
Titration dose increments, (*n* = 2)	
mcg/kg/min (*n* = 1)	5
mg/kg/hr (*n* = 1)	0.1
Titration time, minutes (*n* = 2)	30
Titration endpoints, *n* (%), (*n* = 11)^†^	
Sedation score	4
Pain score	2
Unknown	7

*Ketamine fixed dose*	
Ketamine-fixed-rate CI, *n* (%)	13 (54)
Initial rate, dose, median (IQR)	
mcg/kg/min (*n* = 6)	6.0 (3.5–9.3)
mg/kg/hr (*n* = 7)	0.6 (0.5–1.1)

^*∗*^All doses converted to mg/kg/hr. ^†^Some patients had both a pain and sedation score endpoint. IQR: interquartile range; CI: continuous infusion.

**Table 4:** Clinical outcomes associated with continuous infusion ketamine (*n* = 24).

Clinical outcome	Value
Length of stay, days, median (IQR)	
ICU	22 (5–40)
Hospital	27 (7–68)
Duration of MV, days, median (IQR) (*n* = 22)	15 (6–36)
Mortality, *n* (%)	
ICU	1 (4)
Hospital	1 (4)
Discharge disposition (*n* = 23), *n* (%)	
Home/correctional facility	17 (74)
SNF/long-term care/rehab center	3 (13)
Transfer to another hospital	3 (13)

ICU: intensive care unit; MV: mechanical ventilation; SNF: skilled nursing facility.

## Data Availability

The data supporting the findings of this study are included within the article and its supplementary materials. Due to the nature of the research study, the raw data are not available for privacy/legal reasons and within compliance of institution's Investigational Review Board approvals.

## Supplementary Materials

Supplementary Materials Table S1: institutional demographics. Table S2: individual pediatric patients receiving continuous infusion ketamine. Table S3: continuous infusion ketamine daily doses, minimum/maximum doses, and volume infused.
