# Supplementary material for: Multicenter Retrospective Review of Ketamine Use in Pediatric Intensive Care Units (Ketamine-PICU Study)
Source: Crit Care Res Pract. 2024 Jul 27;2024:6626899. doi: 10.1155/2024/6626899 (PMC11300064; doi:10.1155/2024/6626899)
Supplement: Supplementary Materials — Table S1: institutional demographics. Table S2: individual pediatric patients receiving continuous infusion ketamine. Table S3: continuous infusion ketamine daily doses, minimum/maximum doses, and volume infused. [file 6626899.f1.docx]

**SUPPLEMENTARY MATERIAL**

Table S1: Institutional Demographics (n=4)

| Demographics | n (%) |
| --- | --- |
| Type of hospital  University teaching  Community | 2 (50)  2 (50) |
| Hospital location region*  Northeast  Southeast  Midwest  Southwest  West | 1 (25)  1 (25)  1 (25)  1 (25)  0 (0) |
| Total hospital beds  250-499  500-749  750-1000 | 1 (25)  1 (25)  2 (50) |
| Total ICU beds (excluding neonatal ICU)  51-75  >75 | 1 (25)  3 (75) |
| Pain assessment scale  NRPS  CPOT  BPS  NVPS | 4 (100)  2 (50)  1 (25)  1 (25) |
| Sedation assessment scale  RASS  SAS | 3 (75)  1 (25) |
| Delirium assessment tool  CAM-ICU | 4 (100) |
| ICU admission required for CI ketamine, yes  Non-ICU locations CI ketamine allowed (n=2)  Step-down/PCU  Emergency room  Floor | 2 (50)  2 (100)  2 (100)  2 (100) |
| MV requirement for CI ketamine, yes  Invasive or non-invasive  Invasive only | 2 (50)  2 (50)  2 (50) |
| Titratable nursing administration permitted, yes  State limitations for nursing administration, yes | 4 (100)  2 (50) |
| CI ketamine units  mcg/kg/min  mg/kg/hr  mg/hr | 3 (75)  1 (25)  1 (25) |

Abbreviations: ICU: intensive care unit; NRPS: numerical rating scale 1-10; CPOT: critical care pain observation tool; BPS: behavioral pain scale; NVPS: nonverbal pain scale; RASS: Richmond Agitation and Sedation Scale; SAS: Riker Sedation Agitation Scale; CAM-ICU: confusion assessment method for the ICU; CI: continuous infusion; PCU: progressive care unit; MV: mechanical ventilation

^*^Regions: Northeast [Maine, Massachusetts, Rhode Island, Connecticut, New Hampshire, Vermont, New York, Pennsylvania, New Jersey, Delaware, Maryland]; Southeast [West Virginia, Virginia, Kentucky, Tennessee, North Carolina, South Carolina, Georgia, Alabama, Mississippi, Arkansas, Louisiana, Florida]; Midwest [Ohio, Indiana, Michigan, Illinois, Missouri, Wisconsin, Minnesota, Iowa, Kansas, Nebraska, South Dakota, North Dakota]; Southwest [Texas, Oklahoma, New Mexico, Arizona]; West [Colorado, Wyoming, Montana, Idaho, Washington, Oregon, Utah, Nevada, California, Alaska, Hawaii]

Table S2: Individual Pediatric Patients Receiving Continuous Infusion Ketamine (n=24)

| Age (years) | Sex | Weight (kg) | Admitting Diagnosis | Clinical Characteristics | CI Ketamine Indication | Line/Day of Therapy^*^ | Starting Dose mg/kg/hr  (dose range) | Duration (Days) | Concomitant CI Analgesics/  Sedatives |
| --- | --- | --- | --- | --- | --- | --- | --- | --- | --- |
| 1 | M | 3.7 | Respiratory failure | MV | Analgosedation | First-line/  Day 6 | 0.6 (0.6-0.6) | 3.5 | Midazolam |
| 1 | F | 10.2 | Respiratory failure | MV, NMB | Sedation | First-line/ NA | 1.25 (1-3) | 2.3 | None |
| 1 | F | 6.9 | Seizure | MV, VP | Status epilepticus | NA | 0.6 (0.5-3.7) | 9.0 | NA |
| 1 | M | 6 | Respiratory failure | MV, NMB | Analgosedation | Adjunctive/ Day 7 | 0.5 (0.5-0.9) | 5.2 | Fentanyl Midazolam |
| 1 | M | 4.4 | Respiratory failure | MV, NMB | Sedation | Adjunctive/ Day 3 | 0.5 (0.3-1.1) | 4.4 | Fentanyl |
| 1 | M | 3.9 | Respiratory failure | MV | Analgosedation | Adjunctive/ Day 8 | 1 (1-3) | 1.3 | Fentanyl Midazolam |
| 1 | F | 9.1 | Respiratory failure | MV | Analgosedation | Adjunctive/ Day 1 | 0.5 (0.5-1.5) | 0.4 | Fentanyl Midazolam |
| 2 | M | 12.6 | Respiratory failure | MV, NMB | Sedation | Adjunctive/ Day 1 | 0.5 (0.5-2) | 2.9 | Dexmedetomidine |
| 3 | F | 13.6 | Elective non-cardiac surgery | NA | Analgosedation | Adjunctive/ Day 5 | 0.3 (0.3-4) | 4.9 | Fentanyl  Midazolam Dexmedetomidine |
| 3 | F | 8.8 | Elective non-cardiac surgery | MV | Sedation | Adjunctive/ Day 2 | 1 (1-1.4) | 0.9 | Fentanyl  Morphine Midazolam |
| 5 | M | 17.8 | Septic shock | MV, NMB | Analgosedation | Adjunctive/ Day 7 | 0.6 (0.6-1.2) | 1.7 | Fentanyl Midazolam |
| 6 | F | 23 | Septic shock | MV | Sedation | Adjunctive/ Day 3 | 0.12 (0.12-0.12) | 0.3 | Fentanyl Midazolam Dexmedetomidine |
| 8 | M | 26.8 | Asthma | MV, NMB | Analgosedation | Adjunctive/ Day 1 | 0.42 (0.18-0.42) | 1.9 | Midazolam |
| 8 | F | 44.7 | Septic shock | MV, VP | Analgosedation | First-line/ NA | 0.6 (0.6-0.6) | 0.2 | None |
| 9 | F | 29.5 | Seizure | MV, VP | Status epilepticus | NA | 0.5 (0.5-1) | 14.0 | NA |
| 11 | M | 71.2 | Respiratory failure | MV | Sedation | Adjunctive/ Day 9 | 0.5 (0.5-3) | 13.6 | Morphine  Propofol Dexmedetomidine |
| 11 | F | 40.8 | Asthma | NA | Sedation | Adjunctive/ Day 1 | 1 (0.3-1) | 1.3 | Dexmedetomidine |
| 13 | M | 2 | Respiratory failure | MV, NMB | Analgosedation | Adjunctive/ Day 6 | 2 (3-7) | 1.6 | Fentanyl  Propofol Dexmedetomidine |
| 14 | M | 111 | Respiratory failure | MV, NMB, VP | Analgosedation | First-line/ NA | 0.3 (0.3-0.6) | 3.8 | None |
| 15 | F | 62.2 | Seizure | MV, VP | Status epilepticus | NA | 9 (1-9) | 2.4 | NA |
| 16 | F | 67 | Respiratory failure | MV, VP | Analgosedation | First-line/ NA | 0.2 (0.2-0.2) | 0.1 | None |
| 17 | F | 184.5 | Abdominal perforation | MV | Sedation | Adjunctive/ Day 17 | 0.3 (0.3-0.6) | 2.5 | Fentanyl Midazolam Dexmedetomidine |
| 17 | M | 74.8 | Trauma | MV | Analgesia | Adjunctive/ Day 10 | 0.18 (0.18-0.6) | 4.4 | Morphine Midazolam Propofol |
| 17 | F | 47.4 | Respiratory failure | MV | Analgosedation | Adjunctive/ Day 7 | 0.5 (0.5-3) | 4.3 | Hydromorphone |

M=Male; F=Female; CI: Continuous infusion; MV: Mechanical Ventilation; NMB: Continuous neuromuscular blockade; VP: Continuous vasopressors; NA=Not applicable

^*^Line of therapy indicates if CI ketamine was used as a first-line or adjunctive analgesic, sedative, or analgosedative infusion and day of therapy is the day CI ketamine was started on in relation to the first CI analgesic/sedative the patient received

Table S3. Continuous Infusion Ketamine Daily Doses, Minimum/Maximum Doses, and Volume Infused (n=24)

| Day | First 24H | 25-48H | Day 3 | Day 4 | Day 5 | Day 6 | Day 7 |
| --- | --- | --- | --- | --- | --- | --- | --- |
| n | 24 | 12 | 9 | 5 | 3 | 2 | 2 |
| Cumulative daily dose: mg | 322  (140-880) | 488  (256-1396) | 613  (262-1589) | 613  (539-668) | 613  (424-2870) | 2870  (1741-3998) | 2870  (1741-3998) |
| Daily dose: mg/kg/hr | 0.9  (0.6-1.7) | 0.9  (0.5-1.9) | 0.9  (0.6-3.7) | 0.5  (0.3-3.0) | 3.0  (1.6-3.3) | 3.3  (3.2-3.5) | 3.3  (3.2-3.5) |
| Daily dose: mg/hr | 21  (9-41) | 22  (20-58) | 31  (22-66) | 26  (22-32) | 256  (24-120) | 120  (73-167) | 120  (73-167) |
| Minimum daily dose: mg/kg/hr | 0.6  (0.3-1) | 0.8  (0.3-1.1) | 0.6  (0.3-3) | 0.6  (0.3-3.0) | 3  (1.7-3.4) | 3.4  (3.2-3.5) | 3.4  (3.2-3.5) |
| Minimum daily dose: mg/hr | 10  (4-25) | 22  (11-58) | 31  (22-55) | 26  (22-33) | 26  (24-120) | 120  (73-167) | 120  (73-167) |
| Maximum daily dose: mg/kg/hr | 1.0  (0.6-2.0) | 0.9  (0.6-2.3) | 2  (0.6-3.0) | 0.6  (0.3-3) | 3.0  (0.7-3.4) | 3.4  (3.2-3.5) | 3.4  (3.2-3.5) |
| Maximum daily dose: mg/hr | 23  (12-42) | 25  (22-58) | 31  (25-67) | 26  (22-33) | 26  (24-120) | 120  (73-167) | 120  (73-167) |
| Volume infused: mL | 161  (53-281) | 287  (59-446) | 255  (61-347) | 269  (61-513) | 118  (90-315) | 287  (174-400) | 287  (174-400) |

Data presented as median (IQR)

Abbreviations: H: hour
